# MAC30 Knockdown Inhibits Proliferation and Enhance Apoptosis of Gastric Cancer by Suppressing Wnt/*β*-Cateninsignaling Pathway

**DOI:** 10.1155/2020/6358685

**Published:** 2020-08-21

**Authors:** Xiaohong Wu, Yong Zhang, Junping Guo, Xun Yan, Li Shen, Jianping Zhou, Jun Zhao, Min Zhuang, Zhihong Cao

**Affiliations:** ^1^Department of General Surgery, the Affiliated Yixing Hospital of Jiangsu University, Yixing 214200, China; ^2^Department of Pharmacy, the Affiliated Yixing Hospital of Jiangsu University, Yixing 214200, China; ^3^Department of General Surgery, Binhai County People's Hospital of Jiangsu Province, Binhai 224500, China; ^4^Disinfection Supply Center, the Affiliated Yixing Hospital of Jiangsu University, Yixing 214200, China; ^5^Department of Gastrointestinal Surgery, the Affiliated Yixing Hospital of Jiangsu University, Yixing 214200, China; ^6^Department of Chest Surgery, Liyang People's Hospital, Liyang 213371, China; ^7^Department of Radiology, the Affiliated Yixing Hospital of Jiangsu University, Yixing 214200, China; ^8^Deputy Chief Inspector of Laboratory Division, the Affiliated Yixing Hospital of Jiangsu University, Yixing 214200, China; ^9^Department of Gastroenterology, the Affiliated Yixing Hospital of Jiangsu University, Yixing 214200, China

## Abstract

Gastric cancer is one of the most frequently diagnosed cancer and poses a serious threat to health system in the world. Upregulation of meningioma-associated protein (MAC30) has been found in many solid tumors and can regulate the proliferation, differentiation, and apoptosis of different tumor cells. Quantitative polymerase chain reaction (qPCR) was used to detect the expression of MAC30 in 68 patients with gastric cancer and their adjacent tissues. Lentiviral vector pGCSIL-shMAC30-GFP of the RNA interference (RNAi) of the MAC30 gene was transfected into gastric cancer BGC-823 cell line and the expression of lentivirus label protein GFP was observed via fluorescence microscope, while cell proliferation and apoptosis were determined with flow cytometry and MTT assay, respectively. Also, related protein expressions on Wnt/*β*-catenin signaling pathway were analyzed by Western blot method. The expression of MAC30 was abnormally elevated in gastric cancer tissues, while interfering of its expression could significantly inhibit the proliferation of gastric cancer BGC-823 cell line. However, the promotion of apoptosis by mitochondrial pathway was mediated by Bax/Bcl-2 upregulation. Present work showed the effect of downregulated MAC30 expression on proliferation and apoptosis of gastric cancer cell through Wnt/*β*-catenin signaling pathway. Thus, this investigation provides an experimental basis for future development of chemotherapeutic agent on gastric cancer.

## 1. Introduction

Gastric cancer is the fifth most commonly diagnosed cancer and the third leading cause of cancer death worldwide with over 1.03 million new cases with about >782,000 deaths annually predicted in 2018, representing close to 1 in 12 deaths globally [1]. In the past few decades, many studies have shown that gastric tumorigenesis is related to the genetic and epigenetic alteration, so identification of oncogenes and regulatory mechanisms of oncogenic pathways is greatly significant for the treatment of gastric cancer [2].

Meningioma-associated protein (MAC30), also named (TMEM97) transmembrane protein 97, a member of the insulin-like growth factor-binding protein family, was firstly designated as tumor suppressor gene in view of its upregulation in meningiomas [3]. It has been found that MAC30 is differentially expressed in various tumors and can regulate the proliferation, differentiation, and apoptosis of several tumor cells. Expression of MAC30 mRNA was elevated in colorectal cancer, human pure squamous cell lung cancer (SQCLC), and gastric and breast cancers, while it is lowly expressed in pancreatic and renal cancers [4–6].

In the study by Xu [7], it was shown that MAC30 knockdown expression could inhibit AKT phosphorylation, CyclinB1, and WAVE2 activities which caused interference in the proliferation, migration, and invasion of human gastric cancer cell lines (BGC-823 and AGS) through suppression of AKT signaling pathway. Furthermore, downregulation of MAC30 expression inactivated *β*-catenin, survivin, and cyclin D1 via suppression of Wnt/*β*-catenin and PI3K/Akt signaling pathways which affected the invasion and EMT of breast cancer MCF-7 and MDA-MB-231 cells [8].

Wnt/*β*-catenin signaling pathway has been implicated to play a major role in many tumorigenesis and participated in the process of regulation, differentiation, proliferation, and apoptosis, while blocking and alteration of this pathway are beneficial for cancers therapy [9]. However, the mechanism by which MAC30 regulates the proliferation and apoptosis of gastric cancer cells through the Wnt/*β*-catenin signaling pathway has not been reported yet.

Therefore, this study aims to explore the mechanism of effect of Wnt/*β*-catenin signaling pathway on the proliferation and apoptosis of gastric cancer BGC-823 cells by interfering with the expression of MAC30, so as to provide a potential therapeutic target for clinical treatment of gastric cancer.

## 2. Materials and Methods

### 2.1. Clinical Specimen and Data Collection

Around 0.6 cm fresh gastric adenocarcinoma specimens and same size of their adjacent tissues (cut out 2 cm away from tumor margin) were obtained from 68 gastric cancer patients (comprising 42 males and 26 females, age ranged from 42~76, averagely 59.54 ± 5.72 years) were recruited between April 2017 and June 2019 (at Yixing people's hospital). The normal gastric mucosa was also collected at the same time. After the tumor resection, tissue samples were immediately put into cold storage tube for liquid nitrogen preservation. Sample retention was performed carefully to prevent cross contamination. The experiment of this study was approved by the hospital ethics committee, and all the patients involved in the study signed the informed consent and patients who had received preoperative adjuvant radiotherapy or chemotherapy were excluded from this research. Diagnosis was confirmed by pathological examination, and all specimens were removed in our hospital.

### 2.2. Cell Origin and Culture

Gastric cancer BGC-823 cell line was purchased from Shanghai Institute of Cell Biology, Chinese Academy of Sciences, and cryopreserved in liquid nitrogen. After taking out the BGC-823 cell line, it was dissolved in a 37°C water bath; consequently, DMEM (Dulbecco's Modified Eagle Medium) medium containing 10% fetal bovine serum (FBS) was added. Then, it was centrifuged to remove the supernatant, while cell culture solution was added to suspend the cells prior to inoculation in a culture flask. The cells were cultivated at 37°C and 5% CO_2_, and when the degree of fusion reached 80%, it was digested with 0.25% trypsin and passaged in complete medium. The passages were consistent, and the cells were in the log phase for subsequent studies.

### 2.3. Reagents and Instruments

Fetal bovine serum, trypsin, DMEM medium, penicillin, and streptomycin were all purchased from Gibco Co. Ltd., USA, while Wnt/*β*-catenin signaling pathway inhibitor DKK-1 was obtained from Cell Signaling Technology, USA. Lipofectamine™ 2000 was acquired from Invitrogen Co. Ltd., USA. Rabbit antihuman MAC30 monoclonal antibody, rabbit antihuman Wnt3*α*, rabbit antihuman phosphorylated GSK-3*β* (p-GSK-3*β*) antibody, rabbit anti-*β*-catenin, rabbit anti-GAPDH, and horseradish peroxide-labeled goat anti-rabbit IgG (HRP Goat anti-rabbit IgG) antibody were bought from Abcam Co., Ltd., USA. The JC-1, MTT kit, TUNEL apoptosis detection kit, BCA protein assay kit, TRIzol reagent, and PrimeScrip reverse transcription kit were procured from Shanghai Jingke Chemical Technology Co., Ltd. Mitochondria membrane potential detection kit was obtained from R & D Co., Ltd., USA, while the cell incubator was provided by Shanghai Santeng Instrument Co., Ltd. All the interference plasmids involved in this experiment were constructed by Shanghai Jikai gene Chemical Technology Co., Ltd.

## 3. Methods

### 3.1. RNA Isolation and Quantitative Polymerase Chain Reaction (qPCR)

The total RNA was extracted by Trizol reagent and transcribed into complementary DNA (cDNA) according to PrimeScrip reverse transcription kit, following manufacturers' instructions. The qPCR reaction system was configured using SYBR Premix Ex Taq. The reaction was performed under the following condition: initial denaturation at 95°C for 10 min, then 95°C 10 s, 60°C 30 s, 72°C 10s, 40 cycles; 5 s at 95°C, 1 min at 60°C, 30 s at 95°C, respectively. U6 was used as internal reference and the primer sequences were as stated below: U6 upstream, 5′-CTCGCTTCGGCAGCACA-3′; U6 downstream, 5′-AACGCTTCACGAATTTGCGT-3′; MAC30 upstream, 5′-AACCCTTGTAACTGTCTCCC-3′; MAC30 downstream, 5′-CGAGGTGTGCAGGGTC-3′. The relative expression was calculated by 2^-*△△*CT^ method. Each sample was independently repeated 3 times.

### 3.2. Synthesis of Interference Nucleotide Sequence

HEK293T cells were resuscitated with DMEM medium containing 10% fetal bovine serum, 100 IU/mL penicillin, and 100 *μ*g/mL streptomycin and cultured at 37°C and CO_2_ 50 mL/L. To design and synthesize the nucleotide sequence of siRNA targeting MAC30 gene, we firstly constructed the lentiviral vector pGCSIL-shMAC30-GFP and the empty plasmid pGCSIL-shCON-GFP, which was consequently transformed into the competent cell DH5 *α*. After that, the positive clone for amplification and sequencing was selected; then, we expanded the culture and purified the plasmid vector. The DNA of lentivirus packaging system was transfected into HEK293T cells according to the instructions of lipofectamine TM2000. After 8 h of transfection, the culture medium was replaced and cultured for another 48 h, while the cell supernatants rich in lentiviral particles were collected, concentrated by filtration and centrifugation. Next, the virus concentrates were collected and stored at -80°C after titer determination, respectively, as lentivirus vector and empty body groups.

### 3.3. Transfection and Construction of Cell Lines

The concentration of BGC-823 cell line was adjusted to 1 × 10^6^ cells/mL. Next, 2 mL of BGC-823 cell line was inoculated into 6-well plate and cultured overnight. According to the virus infection ratio value of 50 : 1, two groups of active virus particles (lentivirus vector and empty body groups) were taken to infect target cells. After 8 h, the culture medium was replaced with new ones and culture was continued for 48 h. Transfection cell lines were obtained through drug screening as the interference group and the negative group, while the expression of the green fluorescent label was observed under a fluorescence microscope.

### 3.4. MTT Assay

After 48 h of transfection, the two groups of cells in section 1.4.3 were inoculated into 96 well plates by adjusting the cell concentration to 3 × 10^4^ cells/mL. Aliquot (200 *μ*L) of cell suspension was added into each well and cultured at 37°C and 5% CO_2_ for 24, 48, and 72 h accordingly. After that, 10 *μ*L of MTT reagent was added and incubated at 37°C for 4 h. The absorbance (A) was measured at 490 nm, while the cell proliferation rate was calculated as following equation. Meanwhile, untransfected BGC-823 cells were used as blank control.

(1) $$\begin{array}{c} \text{Cell Proliferation Rate}=\frac{Transfection\;group\;A}{Blank\;group\;A}\times 100\% \end{array}$$

### 3.5. Flow Cytometry Detection

After transfecting the two groups of cells in section 1.4.3 for 48 h, the cells were washed with precooled PBS, and 300 *μ*L of buffer solution was added to suspend the cells. Then, the cell concentration was adjusted to 1 × 10^6^ cells/mL, and 100 *μ*L was taken and placed in a flow tube, while 5 *μ*L each of Annexin V-FITC and PI were added, mixed, and incubated for 15 min at room temperature. Finally, 400 *μ*L PBS was added, and apoptosis was detected on the machine. Meanwhile, untransfected BGC-823 cells were used as blank controls.

### 3.6. Western Blot Analysis

After 48 h of transfection, the BGC-823 cells were added to appropriate radioimmunoprecipitation assay (RIPA) lysate, lysed for 30 min, and centrifuge (12000/min) at 4°C for 10 min, and the supernatant was collected. Next, the protein concentration was measured using the bicinchoninic acid (BCA) kit. Loading buffer was mixed and denatured at 100°C for 5 min, before it was added to the prepared sodium dodecyl sulfate SDS-polyacrylamide gel electrophoresis PAGE gel (5% concentrated gel, 10% separation gel) loading wells (each well was 25 *μ*L). Then, the voltage was adjusted to 60 V when concentrating the gel, while separation gel voltage was 120 V. After completion, the gel was removed, and the film was transferred at 4°C for 1.5 h. The polyvinylidene difluoride (PVDF) membrane was blocked with 5% skimmed milk powder for 2 h. The Wnt2, p-GSK-3*β*, and *β*-catenin antibodies were added, and the film was washed at 4°C overnight. Then, HRP Goat anti-rabbit IgG was added. After incubation at 37°C for 2 h, ECL was added for development. Images were collected using an automatic gel imaging system, and GAPDH was used as an internal reference to analyze protein levels. Meanwhile, untransfected BGC-823 cells were used as blank controls.

### 3.7. Detection of Mitochondrial Potential Changes

According to the instructions of JC-1 mitochondrial membrane potential detection kit, the experimental steps were completed in turn. After the transfected cells were cultured for 48 h, the color level of the cell was observed at different wavelengths, including red fluorescence: excitation wavelength (490 nm), emission wavelength (580 nm); green fluorescence: excitation wavelength (490 nm), and emission wavelength (520 nm). The fluorescence images were collected by computer, and the red green fluorescence was overlapped by Image J software. The ratio of the red and green fluorescence density of the synthesized fluorescence was used as the membrane potential expression level value.

### 3.8. Effect of DKK-1 on Proliferation and Apoptosis of Gastric Cancer Cells

DKK-1, a 15 *μ*mol/L (Wnt/*β*-catenin signal pathway inhibitor), was added to the untransfected BGC-823 cells as the inhibitor and untreated groups. After 48 h, MTT method was used to detect cell proliferation, while flow cytometry was used to detect apoptosis. Western blotting was used to detect the expression of Wnt2, p-GSK-3*β*, *β*-catenin, Bax, and bcl-2 protein.

### 3.9. Statistical Analysis

Data were processed using SPSS16.0 software. Single-factor analysis was used for comparison between multiple groups, while independent paired *t*-test was used for pairwise comparison. A *P* < 0.05 was regarded as statistically significant.

## 4. Results

### 4.1. Expression of MAC30 in Gastric Cancer and Its Adjacent Tissues

**Figure 1:**
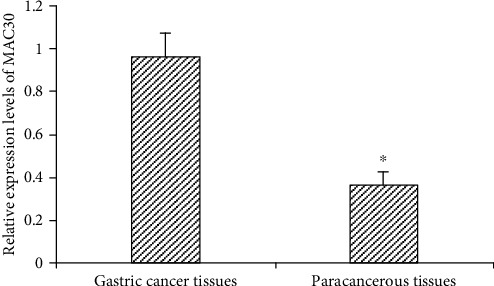
Relative expression of MAC30 in gastric cancer and its adjacent tissues (^∗^*P* < 0.05, compared with gastric cancer group).

The qPCR test results showed that the relative expression levels of MAC30 in the diseased tissues and corresponding adjacent tissues of 68 gastric cancer patients were 0.96 ± 0.11 and 0.36 ± 0.06, respectively, while the differences were statistically significant (*t* = 39.487, *P* < 0.05) as shown in Figure 1. Overall, this result suggests that MAC30 is expressed in gastric cancer and its adjacent tissues, while the expression was increased in gastric cancer.

### 4.2. Establishment of Gastric Cancer BGC-823 Cell Line Transfected with Lentivirus

**Figure 2:**
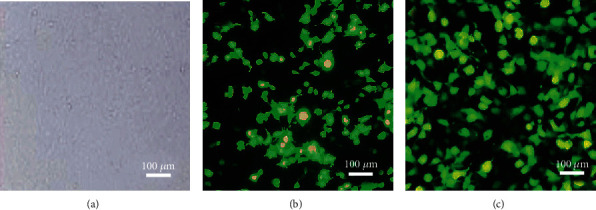
Expression of green fluorescent tag protein in gastric cancer cell lines of each group. (a) Blank group. (b), Negative group. (c), Interference group.

The gastric cancer BGC-823 cells infected with lentivirus were screened to obtain MAC30-expressing cell lines and empty vector cell lines as interference and negative groups, respectively. Observation under a fluorescence microscope showed that the cell lines in both the negative and the interference groups showed green fluorescence, that is, the expression of the tag protein GFP on the lentivirus expression vector, indicating that the infection was successful, while the blank group cells did not display green fluorescence (Figure 2). Importantly, gastric cancer was successfully established in the BGC-823 cell line after transfection with lentivirus.

### 4.3. Expression of MAC30 in Gastric Cancer BGC-823 Cell Line after Transfection with Lentiviral Vector

**Figure 3:**
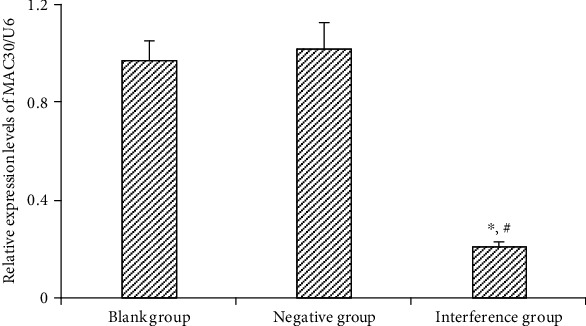
Expression of MAC30 in BGC-823 cell line of gastric cancer detected by qPCR (^∗^*P* < 0.05, compared with blank group; ^#^*P* < 0.05, compared with negative group).

Through qPCR, it was shown that the relative expression of MAC30 in blank and negative groups was 0.97 ± 0.08 and 1.02 ± 0.11, respectively, albeit no significant (*P* > 0.05) difference observed between the two groups; while the relative expression (0.21 ± 0.02) of MAC30 in the interference group was significantly (*P* < 0.05) lower than that in the blank and negative groups (Figure 3). Notably, these results suggest that the expression of MAC30 was decreased in gastric cancer BGC-823 cell line after transfection with lentiviral vector.

### 4.4. Effect of MAC30 Lentivirus on the Proliferation of Gastric Cancer BGC-823 Cell Line

**Figure 4:**
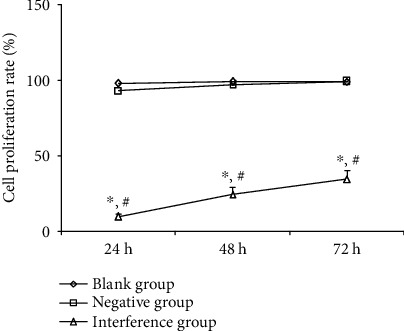
Effect of interference with MAC30 on the proliferation of gastric cancer BGC-823 cell line (^∗^*P* < 0.05, compared with blank group; ^#^*P* < 0.05, compared with negative group).

Based on MTT assay, no statistical difference (*P* > 0.05) was observed in the cell proliferation rates during 24, 48, and 72 h of investigation between the blank and the negative groups. Meanwhile, the cell proliferation rates of the interference group were 9.83 ± 1.41% (24 h), 24.45 ± 4.72% (48 h), and 34.66 ± 5.46% (72 h), amid being significantly (*P* < 0.05) lower than the blank and negative groups at the same time point (Figure 4). Altogether, this finding implies that MAC30 knockdown may inhibit cell proliferation of gastric cancer BGC-823 cell line.

### 4.5. Effect of MAC30 Lentivirus Vector on Apoptosis of Gastric Cancer BGC-823 Cell Line

**Figure 5:**
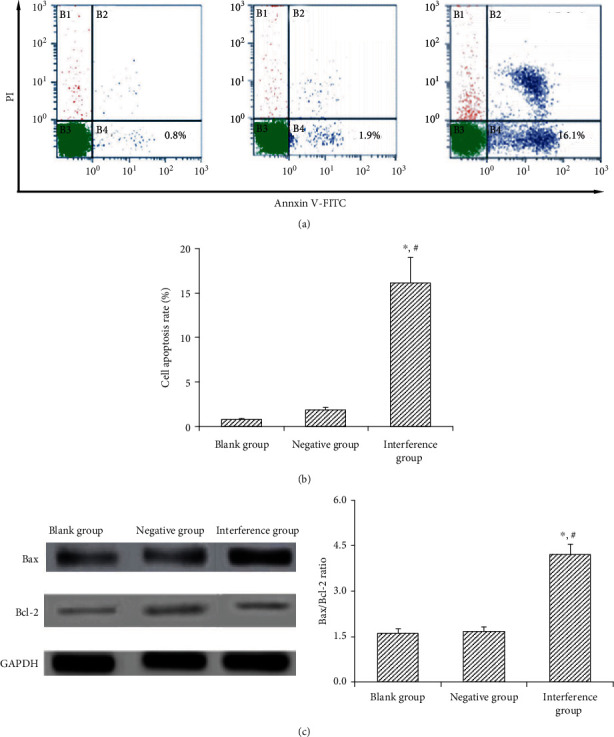
Effect of interference with MAC30 on apoptosis of gastric cancer BGC-823 cell line. (a) TUNEL test chart. (b) Cell apoptosis rate. (c) Ratio of Bax and Bcl-2 protein expression (^∗^*P* < 0.05, compared with blank group; ^#^*P* < 0.05, compared with negative group).

TUNEL apoptosis detection test showed that the apoptosis rate of blank and negative groups were (0.81 ± 0.11)% and (1.88 ± 0.31)%, respectively, which was statistically not significant (*P* > 0.05). Notably, the apoptosis rate of interference group was (16.13 ± 2.89)%, which was significantly (*P* < 0.05) higher than that of blank and negative groups. Also, no significant (*P* > 0.05) difference was observed in Bax/Bcl-2 ratio between the blank (1.62 ± 0.15) and negative groups (1.68 ± 0.16), while that of the interference group (4.21 ± 0.35) was markedly (*P* < 0.05) higher than that of the blank and negative groups (Figure 5). Collectively, these results suggest that knockdown of MAC30 may promote induction of apoptosis in gastric cancer BGC-823 cell line.

### 4.6. Effect of MAC30 Lentiviral Vector on Expression of Wnt/*β*-Catenin Signaling Pathway Related Proteins

**Figure 6:**
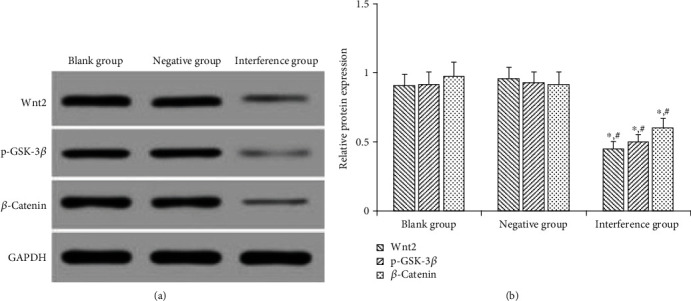
Effect of interference with MAC30 on expression of Wnt/*β*-catenin signaling pathway-related proteins. (a) Western blotting detection electrophoresis. (b) Relative expression of Wnt2, p-GSK-3*β*, *β*-catenin (^∗^*P* < 0.05, compared with blank group; ^#^*P* < 0.05, compared with negative group).

Consistent with previous studies [10, 11], we used Western blot to detect the expression of Wnt2, p-GSK-3*β*, and *β*-catenin to evaluate the effect of MAC30 on Wnt/*β*-catenin signaling pathway in this study. The expression of Wnt2, p-GSK-3*β*, and *β*-catenin protein was observed to be statistically insignificant (*P* > 0.05) between the blank and negative groups, while that of the interference group was substantially lower (*P* < 0.05) than that in the blank and negative groups (Figure 6). Overall, this finding indicates that inhibition of MAC30 may suppress the expression of Wnt/*β*-catenin signaling pathway related proteins.

### 4.7. Effect of MAC30 Lentiviral Vector on Mitochondrial Potential of Gastric Cancer BGC-823 Cell Line

**Figure 7:**
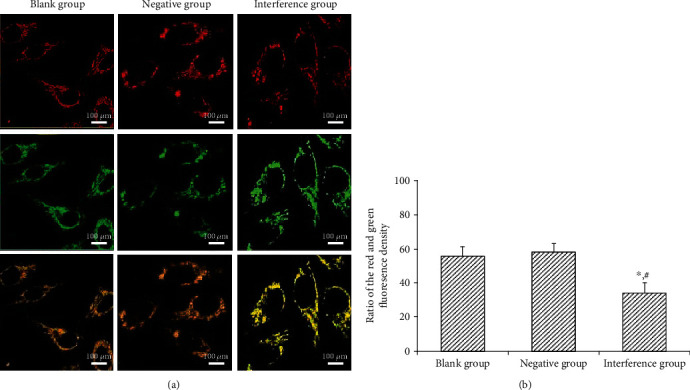
Observation of cell mitochondrial membrane potential. (a) Fluorescence images. (b) Ratio of the red and green fluorescence density (^∗^*P* < 0.05, compared with blank group; ^#^*P* < 0.05, compared with negative group).

After the transfected cells were cultured for 48 h and treated with JC-1, the mitochondrial membrane potentials of the cells in the blank and negative groups were higher (the ratio of red fluorescence to green fluorescence was higher), albeit no significant (*P* > 0.05) difference between the groups. However, the mitochondrial membrane potential of the interference group decreased significantly (*P* < 0.05) compared with the blank and the negative groups (Figure 7). As indicated in the above results, the decrease of the mitochondrial potential of BGC-823 cell line could be achieved by inhibiting MAC30.

### 4.8. Effect of Wnt/*β*-Catenin Signaling Pathway Inhibitor DKK-1 on Proliferation and Apoptosis of Gastric Cancer Cell Line

**Figure 8:**
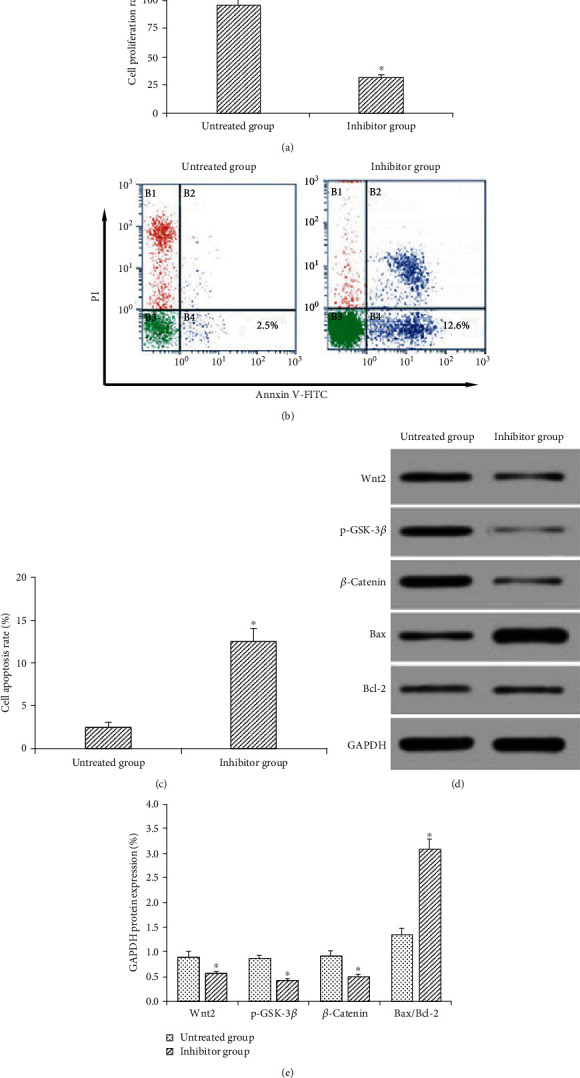
Effects of Wnt/*β*-catenin signaling pathway inhibitor DKK-1 on the proliferation and apoptosis of gastric cancer cell lines. (a) Cell proliferation rate. (b) TUNEL detection chart. (c) Apoptosis rate. (d), Western blotting detection electrophoresis chart. (e) Protein expression (^∗^*P* < 0.05, compared with the untreated group).

DKK-1, 15 *μ*mol/L Wnt/*β*-catenin signal pathway inhibitor, was added to gastric cancer cells for 48 h to detect cell proliferation and apoptosis, as well as Wnt2, p-GSK-3*β*, and *β*-catenin protein expressions. The results showed that the proliferation rate and the expressions of Wnt2, p-GSK-3*β*, and *β*-catenin in the treated group were significantly (*P* < 0.05) lower than those in the untreated group. Apoptosis rate and Bax/Bcl-2 protein ratio were obviously (*P* < 0.05) higher in the treated group than those in the untreated cohort (Figure 8). Collectively, the inhibition of Wnt/*β*-catenin signaling pathway may decrease cell proliferation and increase apoptosis in gastric cancer BGC-823 cell line.

## 5. Discussion

Gastric cancer is a complex, molecularly heterogeneous disease, wherein its pathogenesis is related to the abnormal expression of oncogenes [12] and tumor suppressor genes [13] in the dysregulation of canonical oncogenic signaling pathways such as Wnt/*β*-catenin signaling [14], PI3K/Akt signaling [15], Hippo pathway [16], Notch signaling [17], nuclear factor-*κ*B [18], and p53 pathways [19].

Available literature has established that the activation of the Wnt/*β*-catenin signaling pathway plays a major role in human tumorigenesis, proliferation, differentiation, and invasion by binding Wnt proteins to the Frizzled receptors and LRP5/6 coreceptors through *β*-catenin mediator that initiate a complex signaling cascade [20, 21]. In this regard, inhibition of this pathway has been reported to significantly inhibit the proliferation and migration of breast cancer cells [22] and induce apoptosis in lung cancer by blocking the cell cycle [23].

The classical Wnt/*β*-catenin signaling pathway activation showed that *β*-catenin accumulates in the cytoplasm and then migrates to the nucleus, wherein it interacts with T-cell nuclear factors and lymphokines to initiate downstream transcription of Wnt. Other studies have shown that glycogen synthase kinase-3*β* (GSK-3*β*) can promote the degradation of *β*-catenin, while inhibition of GSK-3*β* has been proven to induce *β*-catenin signaling pathway activation in various tumor models [8].

In recent years, many studies have also posited that the alteration in the expression of several genes could regulate Wnt/*β*-catenin signaling pathway which has been associated with different types of cancers [24]. For instance, epigenetic inactivation of SERP genes allowed constitutive Wnt signaling pathway in colorectal cancer [25], while overexpression of Wnt genes (Wnt 3, Wnt5b, Wnt6, Wnt10a, Wnt14, and Wnt16) activated Wnt signaling pathway in Chronic lymphocytic leukemia [26]. Also, targeted inhibition of cytosolic *β*-catenin attenuated aberrant Wnt signaling pathway, which interfered human tumorigenesis by protein knockdown strategy [27]. Qu [8] firstly reported that downregulated MAC30 expression inhibited the breast cancer cell invasion and epithelial–mesenchymal transition (EMT) by suppressing Akt phosphorylation, *β*-catenin, survivin, and cyclin D1 in PI3K/Akt and Wnt/*β*-catenin signaling pathways.

As one of the members of insulin-like growth factor-binding protein family, MAC30 is highly expressed in meningiomas and is therefore named as such [3]. The expression level of MAC30 varies in many kinds of tumors, namely it is low in pancreatic and renal cancers, but high in carcinomas of the breast, esophageal, gastric, lung, and colon [4, 5, 7]. Moreover, high expression of MAC30 was significantly associated with lymph node metastasis, short survival, and poor prognosis in patients with colon cancer. As an independent prognostic factor, MAC30 played an important role in the development and invasion of colon cancer [26]. At present, the specific role of MAC30 in the regulation of gastric physiology has not been fully defined, but it may be closely related to the differentiation, proliferation, and apoptosis of gastric mucosal cells. Therefore, it is imperative to further explore the molecular mechanism of MAC30 in the regulation of gastric cancer the invasion and metastasis.

Therein, the results showed that the expression of MAC30 in gastric cancer was significantly higher than that in the adjacent tissues, indicating that the increased expression of MAC30 may play a vital role in the occurrence of gastric cancer. RNA interference is a specific gene silencing caused by double-stranded DNA, which has a high inhibition rate and low cytotoxicity to cells. It is the main tool used to study gene function in the present work. In this study, small-molecule RNA was used to interfere with the expression of MAC30 in gastric cancer cell line BGC-823. The results indicated that the interference resulted insignificant lower rate of cell proliferation at 24, 48, and 72 h, while the apoptosis rate was substantially higher than that of the blank and the negative groups after transfection for 48 h. This finding further suggested that MAC30 may play a crucial role in the proliferation and apoptosis of gastric cancer.

Existing studies have found that endogenous apoptosis first occurs in mitochondria, which is generally programmed death caused by Bax gene-mediated protease cascade. Bax, as a major apoptotic gene, destroys the integrity of mitochondrial membrane by binding with mitochondria and promotes its release of apoptotic protein to induce apoptosis. Meanwhile, Bcl-2, as the main antiapoptotic gene, plays a significant role in the outer membrane of mitochondria, which can prevent Bax from binding with mitochondria, via alteration of the permeability of mitochondrial membrane, and thus inhibits apoptosis [28].

In this work, the expressions of the apoptotic protein Bax and the antiapoptotic protein Bcl-2 were detected. The results showed that the ratio of Bax/Bcl-2 protein increased significantly after interfering with the expression of MAC30, which may be an underlying factor for the apoptosis of BGC-823 cells. This phenomenon may be achieved through the Bax/Bcl-2 mediated mitochondrial pathway. Therefore, we further examined the changes in the mitochondrial membrane potential of the cells and found that the mitochondrial membrane potential of the cells in the blank, and the negative groups were higher, while that of gastric cancer cells interfered with MAC30 exhibited a significant decrease in their mitochondrial membrane potential. This finding may verify that mitochondrial pathway might participate in the apoptosis of gastric cancer cells. Xu [7] showed that MAC30 was highly expressed in gastric cancer cell line BGC-823, and after transfection with MAC30 siRNA, MAC30 protein decreased significantly in gastric cancer cells, suggesting that MAC30 may play an important role in the occurrence and development of gastric cancer. Pertinently, overexpression of MAC30 in gastric cancer cells may increase MAC30 protein in gastric cancer tissue and further promote the progress of gastric cancer. Nevertheless, we intend to study this hypothesis in our subsequent experiments. Notwithstanding, other work has shown that overexpression of MAC30 may be involved in the development and invasion of colorectal cancer [29]. Coincidentally, Xiao [6] showed that overexpression of MAC30 is associated with the progression and recurrence of breast cancer.

After interfering with MAC30 expression, we found that Wnt2 and *β*-catenin protein expression was downregulated, while GSK-3*β* phosphorylation was inhibited, suggesting that MAC30 may be related to Wnt/*β*-catenin pathway. In this study, 15 *μ*mol/L Wnt/*β*-catenin signaling pathway inhibitor, DKK-1 was added to gastric cancer cells for 48 h. The results showed that the cell proliferation rate and Wnt2 and *β*-catenin expressions in the inhibitor group were significantly lower than those in the untreated group. The apoptotic rate and Bax/Bcl-2 ratio in the inhibitor group were significantly higher than those in the untreated cohort. The result further suggested that MAC30 might regulate the proliferation and apoptosis of gastric cancer cells through Wnt/*β*-catenin signaling pathway. The specific potential regulation of Wnt/*β*-catenin signaling pathway on MAC30 will be investigated comprehensively in our not-too-distant future experiments.

## 6. Conclusion

In summary, knockdown of MAC30 protein expression in gastric cancer could significantly inhibit the proliferation of gastric cancer BGC-823 cell line. It may also promote apoptosis by upregulating Bax/bcl-2-mediated mitochondrial pathway, which is related to Wnt/*β*-catenin signaling pathway. Therefore, this study might act as the fundamental research for further development of adjuvant chemotherapeutic target on the treatment of gastric cancer.

## Data Availability

The experimental methods and data used to support the findings of this study are included within the article.
